# Red Cinchona

**Published:** 1879-09

**Authors:** 


Red Cinchona—As a remedy for habitual drunkenness is receiving considerable indorsement from English physicians. It is by no means considered a specific, but it is a useful adjuvant to beef-tea and good resolutions.

